# Early skeletal muscle loss in adolescent and young adult cancer patients treated with anthracycline chemotherapy

**DOI:** 10.1002/cam4.6646

**Published:** 2023-10-30

**Authors:** Savannah V. Wooten, Fei Wang, Michael E. Roth, Guanshu Liu, J. Andrew Livingston, Behrang Amini, Susan C. Gilchrist, Michelle Hildebrandt, Eugenie S. Kleinerman

**Affiliations:** ^1^ Department of Pediatrics Research The University of Texas M.D. Anderson Cancer Center Houston Texas USA; ^2^ Department of Sarcoma Medical Oncology The University of Texas M.D. Anderson Cancer Center Houston Texas USA; ^3^ Department of Musculoskeletal Imaging The University of Texas M.D. Anderson Cancer Center Houston Texas USA; ^4^ Department of Cardiology The University of Texas M.D. Anderson Cancer Center Houston Texas USA; ^5^ Department of Lymphoma/Myeloma The University of Texas M.D. Anderson Cancer Center Houston Texas USA

**Keywords:** chemotherapy, clinical observations, Hodgkin's lymphoma, pediatric cancer

## Abstract

**Background:**

Early skeletal muscle loss has been observed in adolescent and young adult (AYA) sarcoma patients undergoing treatment. Identification of individuals within the AYA populace that are at greatest risk of anthracycline‐induced skeletal muscle loss is unknown. Moreover, investigations which seek out underlying causes of skeletal muscle degradation during chemotherapy are critical for understanding, preventing, and reducing chronic health conditions associated with poor skeletal muscle status.

**Methods:**

Computed tomography (CT) scans were used to investigate changes in skeletal muscle of 153 AYA sarcoma and Hodgkin lymphoma patients at thoracic vertebra 4 after anthracycline treatment. Images were examined at three time points during the first year of treatment. In parallel, we used translational juvenile mouse models to assess the impact of doxorubicin (DOX) in the soleus and gastrocnemius on muscle wasting.

**Results:**

Significant reductions in total skeletal muscle index and density were seen after chemotherapy in AYA cancer patients (*p* < 0.01 & *p* = 0.04, respectively). The severity of skeletal muscle loss varied by subgroup (i.e., cancer type, sex, and treatment). Murine models demonstrated a reduction in skeletal muscle fiber cross‐sectional area, increased apoptosis and collagen volume for both the soleus and gastrocnemius after DOX treatment (all *p* < 0.05). After DOX, hindlimb skeletal muscle blood flow was significantly reduced (*p* < 0.01).

**Conclusion:**

Significant skeletal muscle loss is experienced early during treatment in AYA cancer patients. Reductions in skeletal muscle blood flow may be a key contributing factor to anthracycline doxorubicin induced skeletal muscle loss.

## 1 INTRODUCTION

There is a paucity of research on the impact of cancer treatment on musculoskeletal health during and shortly after treatment in adolescent and young adults (AYAs, age 15–39 years at diagnosis) with cancer. Although prior studies have focused on musculoskeletal late effects, up to 20 years after chemotherapy, it is vital to direct attention to muscle degradation prevention via early identification of at‐risk AYAs during early cancer treatment. Our previously published pilot work demonstrated significant reductions in skeletal muscle area and composition at vertebral level T4 after high dose doxorubicin (DOX), which was sustained at 1 year for a cohort of 16 AYA sarcoma individuals.^1^ However, notably, we saw varying degrees of absolute muscle loss and short‐term recovery between patients. Large scale investigations aimed at classifying characteristics of individuals who may be at risk of experiencing comparatively larger skeletal muscle loss are needed.

Skeletal muscle loss is often multifactorial and caused by a variety of factors such as aging, physical inactivity, poor nutrition status, disease state, and disease treatment.^2^, ^3^ Each of these factors independently, as well as synergistically, contributes to skeletal muscle loss via homeostatic imbalance where skeletal muscle degradation is greater than skeletal muscle synthesis.^4^ In AYAs diagnosed with Hodgkin lymphoma or sarcoma, DOX is commonly included in the chemotherapy due to its efficacy.^5^ The relationship of anthracycline cancer treatment, such as DOX, and the development of off target toxicities such as cardiotoxicity, neurotoxicity, hepatotoxicity, gastrointestinal toxicity, and others are well established.^6^, ^7^, ^8^, ^9^, ^10^ However, the area of cancer treatment related skeletal muscle toxicity has received less attention and is poorly understood. Patients treated with anthracyclines have an increased risk of significant skeletal muscle damage, muscle wasting, fatigue, loss of functional capacity, and early mortality.^11^, ^12^

Mouse models have demonstrated that DOX contributes to skeletal muscle toxicity and subsequent skeletal muscle loss through three primary mechanisms; reduction in protein synthesis via down regulation of mTOR, increased skeletal muscle degradation via upregulated myostatin, and mitochondria degradation caused via reactive oxygen species (ROS).^13^ While these direct mechanisms may be able to explain intramuscular degradation, they do not consider the systemic effect of DOX beyond the skeletal muscle. These effects may exacerbate skeletal muscle degradation. One example is the known deleterious effects of DOX on central vasculature.^7^ Given that DOX's impact is systemic and not locally targeted, compromising peripheral vascular function and skeletal muscle blood flow may be an additional mechanism by which DOX contributes to skeletal muscle loss as muscle blood flow is vital for providing essential oxygen and nutrients to maintain skeletal muscle.

The purpose of this retrospective investigation was to classify and quantify early skeletal muscle loss in a large cohort of AYA Hodgkin lymphoma and sarcoma patients during early anthracycline cancer treatment using standard of care computed tomography scans at the level of thoracic vertebrae 4 and identify potential peripheral vascular changes which may additionally contribute to anthracycline‐induced skeletal muscle loss and weakness via mouse model. In addition, we used our recently developed mouse model to investigate the effects of Dox therapy on skeletal muscle blood follow and histology.

## 2 METHODS

### 2.1 Clinical skeletal muscle monitoring model

The internal review board ethics committee of MD Anderson Cancer Center approved the present study which conforms to recognized standards for Declaration of Helsinki; US Federal Policy for the Protection of Human Subjects; or European Medicines Agency Guidelines for Good Clinical Practice. We identified 153 non‐Hispanic White AYA patients who were diagnosed with primary Hodgkin lymphoma or sarcoma between the years of 2000–2016 who received treatment that included DOX. Patients with soft tissue or bone sarcomas were treated with standard of care chemotherapy regimens, surgery, and/or radiation at the discretion of the treating physician. Patients with soft tissue sarcoma subtypes (rhabdomyosarcoma, myxoid liposarcoma, spindle cell sarcoma, synovial sarcoma) received combination chemotherapy including anthracyclines, most often doxorubicin (75 mg/m^2^ per cycle, average cumulative dose 450 mg/m^2^) in combination with ifosfamide with or without vincristine for up to 6 cycles. Patients with osteosarcoma received doxorubicin (75 mg/m^2^ per cycle, average cumulative dose 450 mg/m^2^) in combination with cisplatin with or without the addition of high dose methotrexate. Patients with Ewing's sarcoma received multiagent chemotherapy including combinations of vincristine, doxorubicin, cyclophosphamide, ifosfamide, and/or etoposide. Most patients with Hodgkin lymphoma received between 4 and 6 cycles of the ABVD regimen. Each cycle of chemotherapy included 50 mg/m^2^ of doxorubicin (divided in two doses) for a cumulative anthracycline dose of 200 and 300 mg/m^2^ for patients receiving 4 and 6 cycles, respectively.

Throughout the course of treatment at our institution, the patients had routine computed tomography (CT) scans for disease surveillance. These CT scans were accessed to observe early changes in skeletal during the first year of anthracycline cancer treatment. Indices of skeletal muscle cross‐sectional area (skeletal muscle index, SMI) and composition (skeletal muscle density, SMD) at thoracic vertebrae 4 (T4) at was quantified at 3 time points. Scan 1 was defined as CT imaging prior to anthracycline‐based chemotherapy (baseline). Scan 2 was defined as CT imaging after the completion of initial DOX chemotherapy. Scan 3 was established as 1 year since baseline Scan 1. The erector spinae and transversospinales (EST), trapezius and rhomboid major (TRM), pectoralis major and minor (PMM), and total muscle at T4 (TRM + EST + PMM) were manually segmented and quantified for SMI and SMD using CoreSlicer software.^14^ All participants must have met the following inclusion criteria: (1) age 15–39.99 years at diagnosis; (2) received DOX treatment; and (3) minimum of 2 scans (baseline Scan 1 and Scan 2 or Scan 3).

### 2.2 Mouse model

Balb/c mice (4 weeks, 20–22 g, *n* = 5; NCI with Charles River Laboratories of U.S.) were used in all experiments. Experiments were approved by University of Texas MD Anderson Cancer Center (MDACC) Institutional Animal Care and Use Committee (IACUC) that conform to the Guide for the Care and Use of Laboratory Animals published by the United States National Institutes of Health (N.I.H., Eighth edition, revised 2011).

Mice received phosphate‐buffered saline (PBS control), 2.5 mg/kg (iv by tail vein) DOX (TEVA Parenteral Medicines, Inc. Irvine, CA, U.S.A.) twice a week for 2 weeks. Blood flow in the hindlimb was assessed by ultrasound 24 h after DOX treatment. Mice were euthanized 1 week following therapy completion. The soleus and gastrocnemius muscles were harvested and frozen in OCT, fixed in 10% formalin for evaluation using histology and immunohistochemical analysis.

### 2.3 Skeletal muscle blood flow

Mouse skeletal muscle blood flow was assessed by contrast‐enhanced ultrasound. For acute experiments, contrast‐enhanced ultrasound (CEU) was performed 24 h after treatment (Day 13). The hindlimb blood flow was assessed by three‐dimensional (3D) ultrasound imaging using CEU and 3D color Doppler ultrasound (3D‐CDU) (Vevo 2100; VisualSonics, Toronto, Ontario, Canada). Mice were anesthetized with 2% to 3% isoflurane, and a 21‐gauge 5/8″ needle attached to a 1 mL syringe was placed into the tail vein. During CEU., ultrasound contrast agent microbubbles (Vevo MicroMarker; FUJIFILM VisualSonics, Inc.) were injected through the tail vein catheter according to the manufacturer's instructions.^15^ Before and immediately after the injection of microbubbles, a 3D ultrasound examination of the left hindlimb was performed in nonlinear contrast mode to allow calculation of hindlimb total blood flow volume. Subsequent analyses were performed offline by a sonographer who was blinded to the group's identity. The percentage of contrast agent (PA%) and regions of interest (ROI) were measured by Vevo CQ software, which provides quantitative measurements by computing perfusion parameters by means of a dedicated curve‐fitting algorithm applied on contrast‐uptake kinetics.

### 2.4 Histology and immunohistochemistry

Mouse skeletal muscle tissues were assessed for fiber cross‐sectional area, collagen deposition, and apoptosis using immunofluorescence, and histologic staining. One portion of the soleus and gastrocnemius muscles was fixed in 10% formalin at 4°C overnight before being embedded in paraffin and sectioned for immunohistochemical analysis. Hematoxylin and eosin staining was performed, and sections were analyzed for soleus and gastrocnemius muscle size (diameter of 100 cells per muscle were averaged to obtain 1 value). Masson trichrome staining was performed, and sections were analyzed for collagen deposition of the soleus and gastrocnemius tissue. Collagen volume fraction and perivascular collagen area were measured and quantified using Leica LAS XZD Image Analysis (Leica Imaging Inc, U.S.A.). Apoptosis was assessed using the DeadEndTM Fluorometric TUNEL System (G3250). Images were taken with a Leica DMi8 fluorescent microscope and Leica DC500 digital camera at 200× magnification with Leica Application Suite 4.8.0 software (Leica Imaging Inc, U.S.A.). Exposure settings were adjusted to minimize oversaturation.

### 2.5 Statistical analysis

Linear mixed‐effects model was used for clinical patient analysis to consider the intra‐individual correlation of repeated measurements of skeletal muscle. Difference of the change from Scan 1 to Scan 2 and Scan 3 between subgroups was also estimated in the model. Two‐sided *p*‐value <0.05 was considered significant. Subgroup analysis for sex, cancer type, treatment type, and total anthracycline dosage was conducted to identify individuals which were seen to have skeletal muscle loss. All clinical analyses were performed in SAS 9.4.

Statistical analyses for the mouse studies were performed using GraphPad 9.2.0. Unless otherwise noted in corresponding Methods, comparisons between groups were analyzed by independent two‐tailed *t*‐tests. Two‐factor repeated‐measures ANOVA was used for histology and Masson trichrome staining. Vevo CQ software measured the percentage of contrast agent (PA%), which provides quantitative measurements by computing perfusion parameters using a dedicated curve‐fitting algorithm applied on contrast‐uptake kinetics. A value of *p* < 0.05 was considered statistically significant.

## 3 RESULTS

### 3.1 Clinical patient and computed tomography characteristics

One hundred and fifty‐three patients were included in the study. Sixty‐six (43.1%) were male, and the average patient age at diagnosis was 26 ± 6.3 years. The most common cancer diagnosis was Hodgkin lymphoma (*n* = 120/153), and the remainder of patients were diagnosed with a variety of sarcomas (*n* = 33/153; Table 1). Lower limb was the most common (45.5%) primary tumor location in individuals with sarcoma diagnoses. The remaining sites of sarcoma were variable: Head, 12.1%; Chest, 12.1%; Lung, 3.0%; Upper Limb, 12.1%; Abdomen, 9.1%; and Pelvis 6.1%. Individuals with sarcoma received significantly greater total DOX dose and radiation dose compared to Hodgkin lymphoma patients (*p* < 0.01 for both; Table S1). The average time between skeletal muscle quantification at baseline (Scan 1) and after the completion of primary anthracycline treatments (Scan 2) was 164 ± 63 days. The mean time between Scan 1 and Scan 3 skeletal muscle analysis was 408 ± 148.3 days.

**TABLE 1:** Group demographics and characteristics.

Variable	Means ± SD or *n*
Males/females, *n*	M = 66, F = 87
Age a diagnosis, year	26.7 ± 6.3
Height, m	1.72 ± 0.11
BMI at diagnosis, kg/m^2^	27.2 ± 6.1
Cancer diagnosis	
Hodgkin lymphoma	120
Sarcoma	33
Osteosarcoma	8
Ewin's sarcoma	8
Rhabdomyosarcoma	1
Liposarcoma	5
Spindle cell sarcoma	2
Synovial sarcoma	6
Other (unclassified sarcoma (2), Leiomyosarcoma (1)	3
Computed tomography	
Time between baseline scan and scan 2, days	164 ± 63
Time between baseline scan and scan 3, days	408 ± 148.3
Primary anthracycline, *n*	
ABVD	90
CHOP	3
Dox/Cisplat	6
Dox/Ifosf	12
Vinc/Dox/Ifosf	6
VAdriaC	5
Other	32
Secondary anthracycline, *n*	29
Median total anthracycline dosage, mg/m^2^	300 (75,900)
Additional treatment	
Stem cell transplant	16
Surgery	29
Amputation	4
Radiation, *n* (median Gy)	87 (30.6)

A total of 445 CT scans were analyzed in 153 patients. The majority (69%) of the CTs were dedicated CTs of the Chest or Chest with abdomen and pelvis. The rest (31%) were CT portions of PET/CTs. The Chest portions of the dedicated CTs were 98% in arterial phase with contrast. The CT portions of the contrast‐enhanced PET/CTs were predominantly in venous phase without contrast. Regarding voltage, the majority (97%) of the CTs were acquired at 120kVp. Slice thickness for 96.4% of CT scans was 2.5–3.75 mm.

### 3.2 Computed tomography skeletal muscle change

**FIGURE 1:**
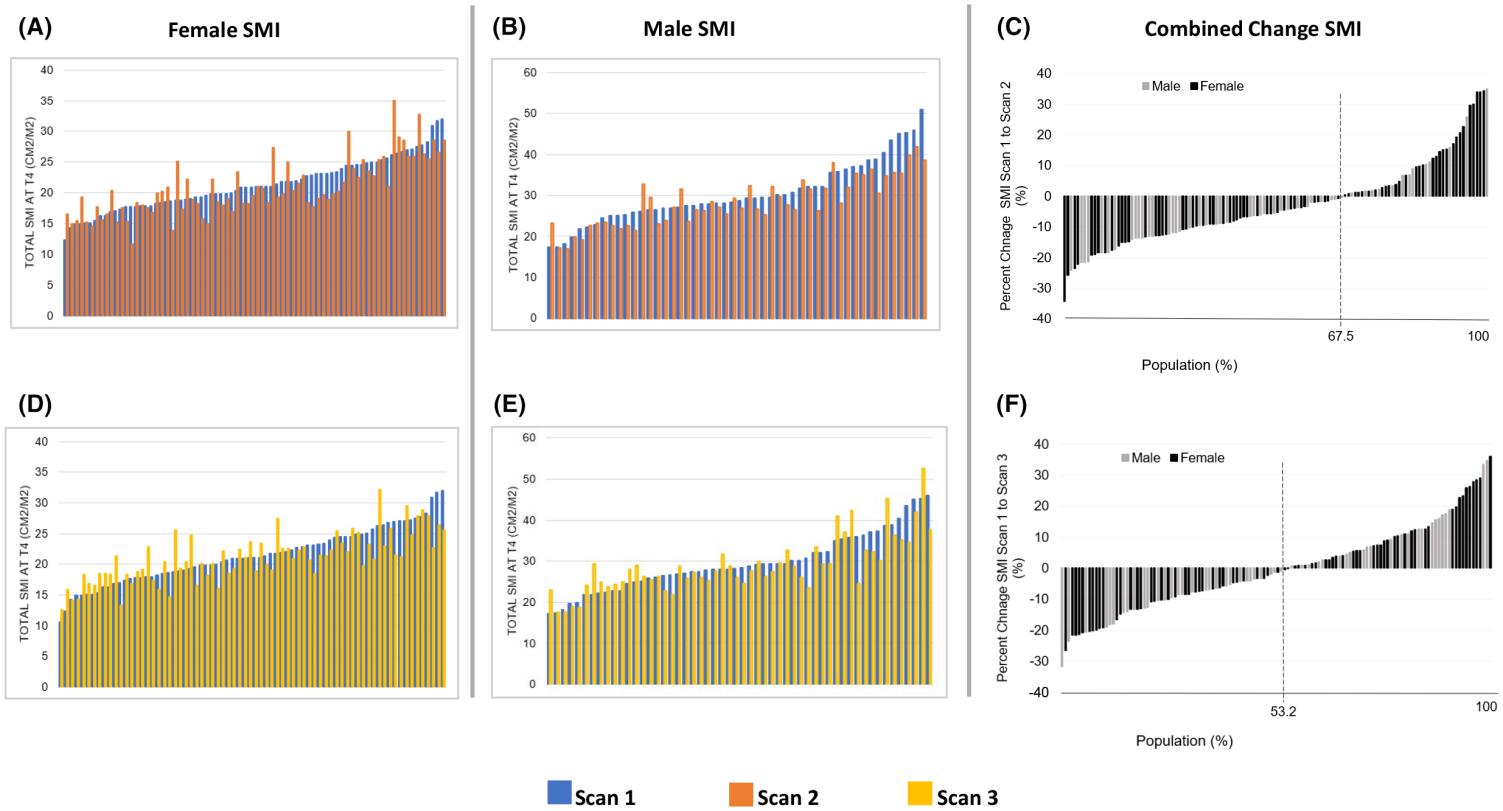
Skeletal muscle index change at T4. (A and D) Absolute skeletal muscle index (SMI) for females. (B and E) Absolute SMI for males. (C) Percent change from Scan 1 to Scan 2 for female and male SMI (F). Percent change from Scan 1 to Scan 3 for female and male SMI.

**FIGURE 2:**
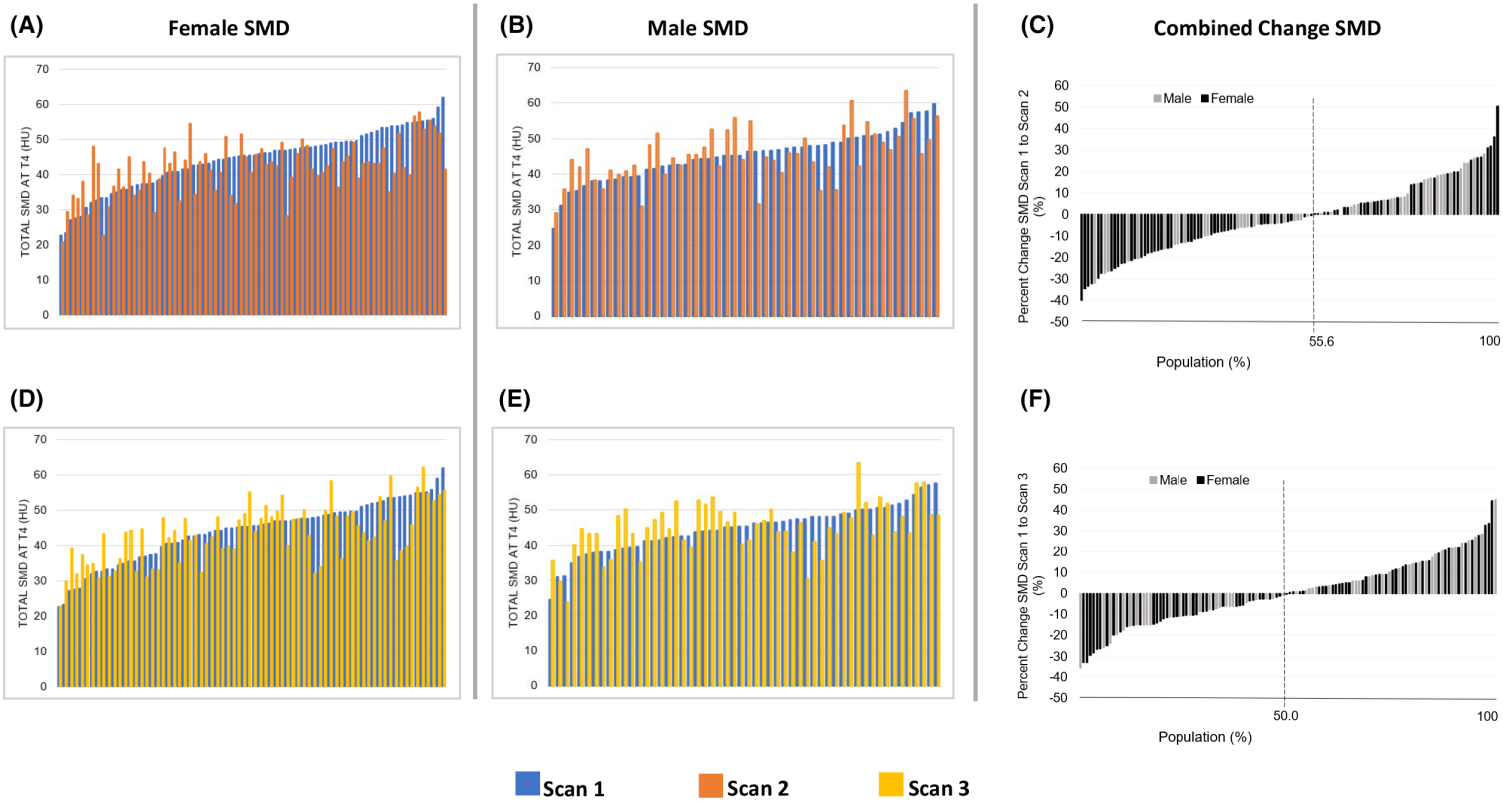
Skeletal muscle density change at T4. (A and D) Absolute skeletal muscle density (SMD) for females. (B and E) Absolute SMD for Males. (C) Percent change from Scan 1 to Scan 2 for female and male SMD (F). Percent change from Scan 1 to Scan 3 for female and male SMD.

AYAs experienced skeletal muscle area and quality reductions at Scan 2, after receiving primary anthracycline treatment (Table 2). Significant SMI loss was seen for PMM (*p* < 0.01) and total muscle (<0.01) at T4. Moreover, significant SMD reduction was seen for EST (*p* = 0.03), PMM (*p* = 0.03), and total muscle (*p* = 0.04) at T4. No significant changes in skeletal muscle index nor density were found at Scan 3, 1 year later when compared to baseline (all *p* > 0.05; Table 2). Although on average the AYA cohort appeared to recover back to baseline skeletal muscle status at 1 year, 53.2% of AYAs were found to fall below baseline T4 skeletal muscle values for SMI at Scan 3 (Figure 1). Moreover, 50% of AYAs were below baseline SMD at Scan 3 (Figure 2).

**TABLE 2:** Summary of skeletal muscle changes during the first year of cancer treatment. Scan 1 (baseline), Scan 2 (after primary anthracycline), and Scan 3 (1 year since Scan 1).

Variable	Total skeletal muscle index (cm^2^/m^2^)					Total skeletal muscle density (HU)				
	Scan 1 mean (SE)	Scan 2 mean (SE)	*p*	Scan 3 mean (SE)	*p*	Scan 1 mean (SE)	Scan 2 mean (SE)	*p*	Scan 3 mean (SE)	*p*
Thoracic vertebrae 4										
Trapezius + rhomboid major	6.5 (0.2)	6.3 (0.2)	0.12	6.4 (0.2)	0.54	44.1 (0.7)	43.7 (0.7)	0.59	44.0 (0.7)	0.94
Erector spinae + transversospinales	3.1 (0.1)	3.0 (0.1)	0.20	3.0 (0.1)	0.15	45.3 (1.0)	43.3 (1.0)	**0.03** *	43.5 (1.0)	0.06†
Pectoralis major + minor	15.4 (0.4)	14.5 (0.4)	**<0.01** *	15.0 (0.4)	0.07†	45.3 (0.8)	43.6 (0.8)	**0.03** *	44.7 (0.8)	0.44
Total T4 muscle	24.7 (0.6)	23.8 (0.6)	**<0.01** *	24.3 (0.6)	0.15	44.7 (0.7)	43.4 (0.7)	**0.04** *	44.1 (0.7)	0.36
Sex										
Male	29.4 (0.7)	27.8 (0.7)	**<0.01** *	28.8 (0.7)	0.236	45.1 (1.0)	45.4 (1.0)	0.70	45.2 (1.0)	0.921
Female	21.3 (0.6)	20.8 (0.6)	0.186	21.0 (0.6)	0.411	44.4 (0.9)	42.1 (0.9)	**<0.01** *	43.4 (0.9)	0.197
Cancer type										
Hodgkin lymphoma	25.1 (0.6)	24.3 (0.6)	**0.022** *	24.9 (0.6)	0.587	44.6 (0.7)	43.6 (0.8)	0.145	44.3 (0.8)	0.605
Sarcoma	24.2 (1.3)	21.9 (1.3)	**<0.01** *	22.4 (1.3)	**0.01** *	45.4 (1.5)	42.8 (1.5)	0.06†	43.6 (1.5)	0.189
Treatment										
No radiation	23.8 (0.9)	22.3 (0.9)	**<0.01** *	23.2 (0.9)	0.257	43.8 (1.0)	43.0 (1.0)	0.41	43.3 (1.0)	0.633
Radiation	25.5 (0.7)	24.8 (0.7)	0.08†	25.1 (0.7)	0.356	45.3 (0.9)	43.8 (0.9)	**0.049** *	44.7 (0.9)	0.413
DOX dosage ≤300 mg/m^2^	27.9 (1.0)	26.2 (1.0)	**<0.01** *	26.5 (1.0)	**0.012** *	44.8 (1.2)	43.2 (1.2)	0.126	44.9 (1.2)	0.962
DOX dosage >300 mg/m^2^	23.3 (0.7)	22.6 (0.7)	0.058†	23.3 (0.7)	0.990	44.6 (0.8)	43.5 (0.8)	0.172	43.7 (0.8)	0.232
3 treatment therapies	23.6 (2.1)	21.7 (2.2)	0.122	22.9 (2.1)	0.514	45.8 (2.3)	44.7 (2.6)	0.660	43.8 (2.4)	0.372

*Note*: 3 treatment therapies (DOX + radiation + surgery).

* Significantly different from baseline Scan 1 *p* < 0.05.

^†^ Approaching significance.

Significant total SMI loss at T4 was seen in male AYAs (*p* < 0.01) when comparing Scan 1 and Scan 2 (Table 2). Significant total SMI loss at T4 was not seen in Female AYAs. However, female AYAs were found to have a significant reduction in skeletal muscle density (*p* < 0.01) for Scan 1 versus Scan 2, whereas male counterparts did not (*p* > 0.05; Table 2). There was no significant difference in SMI nor SMD change by Scan 3 for neither male nor female AYAs (*p* > 0.05 for all) (Table 2). AYAs with a sarcoma as well as Hodgkin lymphoma saw a significant reduction in total SMI at T4 by Scan 2 (*p* < 0.01 and *p* = 0.02, respectively). However, individuals who were diagnosed with sarcoma had significant SMI loss by Scan 3 (*p* = 0.01), whereas those diagnosed with lymphoma appeared to recover back to baseline muscle status (Table 2).

Significant SMI reductions were found for individuals who did not receive radiation (*p* < 0.01) and individuals who received ≤300 mg/m^2^ of DOX (*p* < 0.01). SMI loss was seen at Scan 3 only in individuals who received ≤300 mg/m^2^ of DOX (*p* = 0.012) (Table 2). Individuals who received radiation and individuals who received DOX >300 mg/m^2^ experienced SMI reduction at Scan 2 (*p* = 0.08 & *p* = 0.058, respectively), approaching statistical significance (Table 2). SMD significantly decreased at Scan 2 only in individuals who received radiation (*p* = 0.049) (Table 2). No change in SMD was seen at Scan 3 for any of the treatment related variables (i.e., radiation, no radiation, DOX dosage ≤300 mg/m^2^, DOX dosage >300 mg/m^2^, 3 treatment therapies) (all *p* > 0.05; Table 2). There was no change in SMI and SMD at Scan 2 nor Scan 3 for individuals who received a combination of 3 treatment therapies (DOX, radiation, and surgery; *p* > 0.05 for all, Table 2).

### 3.3 Effect of DOX on mouse skeletal muscle blood flow, morphology collagen deposition and apoptosis

**FIGURE 3:**
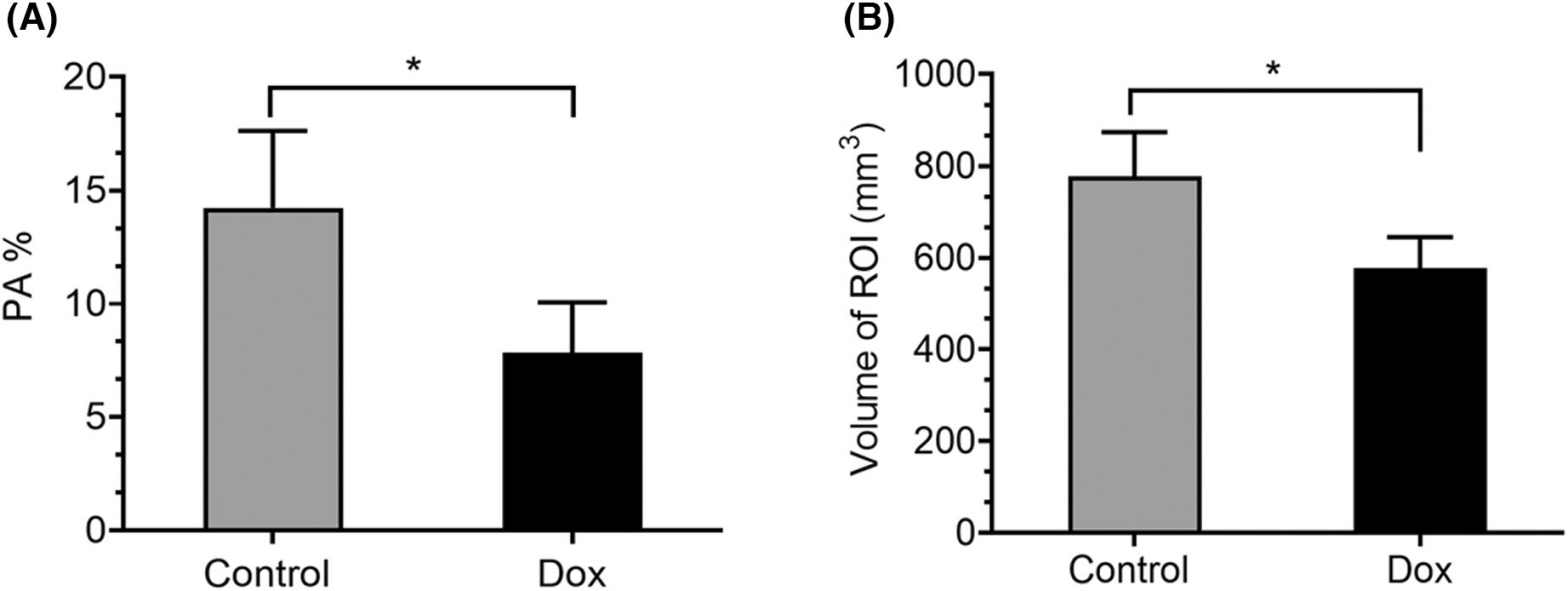
Effect of DOX therapy on blood flow to the hindlimb in mouse model. Mice were treated with PBS or Dox 2 mg/kg twice a week for 2 weeks. Blood flow was assessed using microbubble injection and 3D ultrasound imaging 24 h after Dox therapy. Two parameters were quantified to assess and compare perfusion: (Fig 3A) the change of contrast percentage of pixels exhibiting a signal from the contrast agent (PA%), and (Fig 3B) the volume of blood flow in hindlimb muscle regions of interest (ROI). Values are expressed as the mean ± SEM. *p < 0.05; *n* = 5.

**FIGURE 4:**
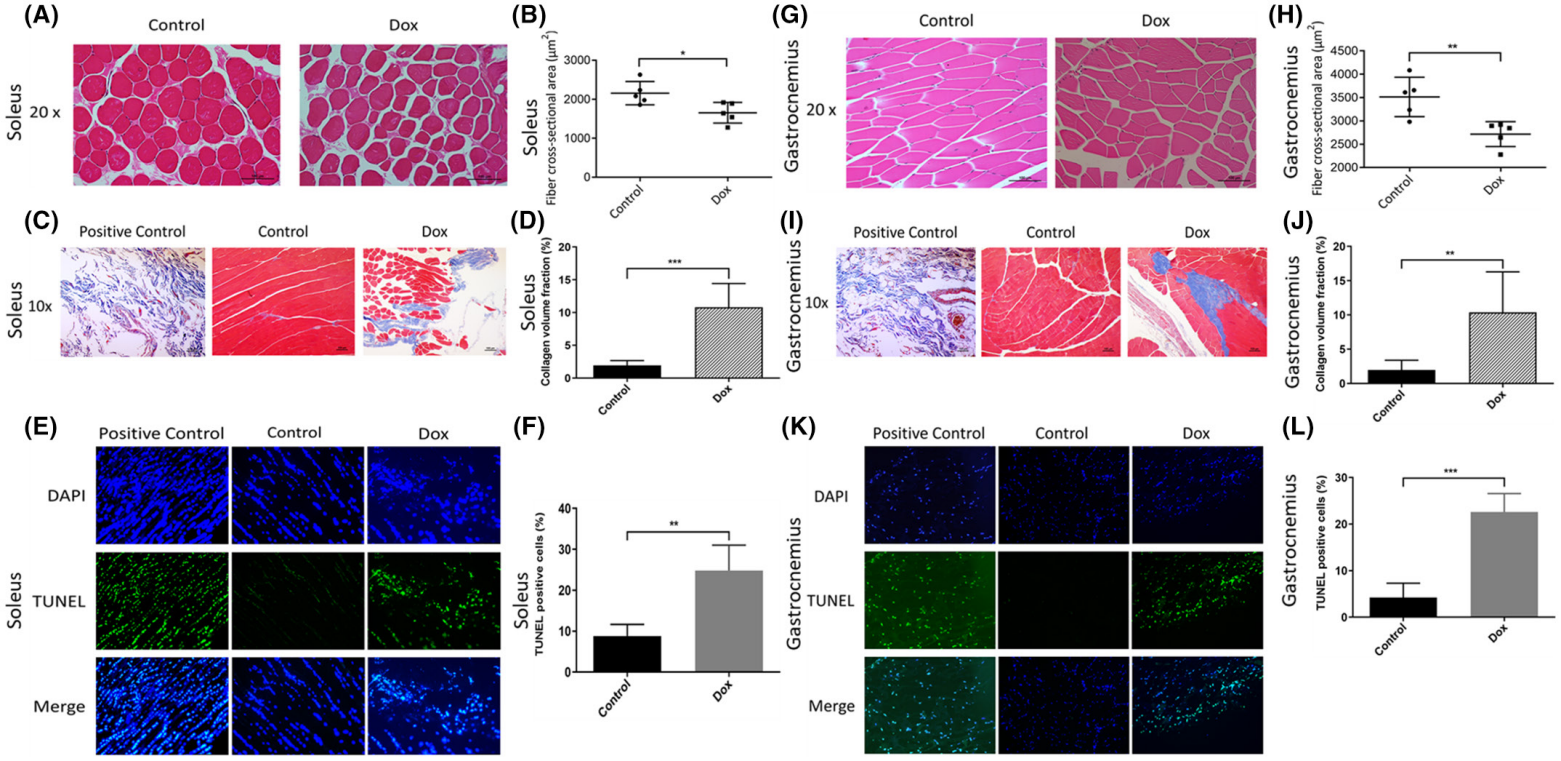
Effect of DOX therapy on morphology, collagen deposition, and apoptosis of soleus and gastrocnemius muscles. Mice were treated as in Figure 3. The soleus and gastrocnemius muscles were harvested 1 week after therapy and analyzed by H&E, Masson trichrome staining and TUNEL to assess morphology, collagen deposition, and apoptosis, respectively, of the soleus (Figure 4A–F) and gastrocnemius muscles (Figure 4G–L). Representative H&E for soleus (A) and gastrocnemius (G). (B, H) Quantitative analysis of the average width of 100 muscle cells/section. Values are the mean ± SEM. *p < 0.05; **p < 0.01. (C, I) Collagen deposition. (D, J) Quantification of collagen deposition by % collagen volume fraction (CVF%). Values are the mean ± SEM. **p < 0.01; ***p < 0.001. (E, K) Representative TUNEL images. Merged images show both DAPI‐and TUNEL‐stained cells (arrows). (F, L) TUNEL‐positive cells per 100 nuclei. Values are the mean ± SEM, *p < 0.05.

Contrast‐enhanced ultrasound was used to assess the effect of DOX therapy on hindlimb muscle blood flow after therapy (Figure 3). The PA% quantified the amount of contrast agent that was delivered to the muscle while the ROI indicates the volume of blood perfusion. There was a significant difference in PA% and ROI in the hindlimb muscles of the DOX‐treated mice compared to the control group. These data indicate that there was compromised blood flow to the muscles of the leg following DOX therapy similar to our finding in cardiac blood flow.^16^ We next evaluated whether DOX therapy affected the histology of the soleus and gastrocnemius muscles (Figure 4). A significant size reduction was seen in both the soleus and gastrocnemius muscles from the DOX‐treated mice 1 week after therapy (Figure 4A,B,G,H). There was also a significant increase in collagen deposition (Figure 4C,D,I,J) and apoptosis (Figure 4E,F,K,L) following DOX therapy in both muscles. The number of TUNEL‐positive nuclei per 100 nuclei was increased in the soleus and gastrocnemius muscles from the DOX‐treated mice. Taken together, these data indicate that DOX therapy significantly affected both blood flow and histology of the hindlimb muscles with the early development of fibrosis.

## 4 DISCUSSION

The present study sought to both classify and quantify early skeletal muscle health changes in AYAs treated with DOX and determine whether DOX therapy affected blood flow to the muscle, as we have demonstrated in cardiac tissue,^17^ as a possible contributing cause to DOX‐induced skeletal muscle loss and patient fatigue. Significant skeletal muscle area and composition reductions were found to occur at T4 during the first year of treatment. Moreover, after classifying the AYA patient population into subcategories by sex, diagnosis, and treatment type, variability in skeletal muscle loss during treatment was seen. Our mouse model demonstrated that DOX therapy significantly reduced skeletal muscle blood flow which may contribute to the decreased size in skeletal muscle fibers and the increased collagen deposition and apoptosis that were observed 1 week after DOX therapy.

After anthracycline treatment, 67.5% of AYAs experienced T4 skeletal muscle area loss and 55.6% experienced reductions in skeletal muscle composition. At 1 year, 53.2% of AYAs were still experiencing a skeletal muscle area deficit and 50% of AYAs saw continuation of composition deficits compared to baseline Scan 1, although neither metric reached statistical significance. However, this finding demonstrates the importance of continual monitoring of individuals rather than utilization of observational group mean differences for determination of skeletal muscle response. In the present case, group means ultimately masked a large portion of the cohort were experiencing significant loss. Approximately, one quarter of individuals experienced skeletal muscle area and composition reductions of ≥10% at 1 year compared to baseline. Future studies should seek to identify, define, and predict individuals early who are at high‐risk for skeletal muscle loss, weakness, and fatigue. Moreover, given that the patient population was an all‐White non‐Hispanic cohort, there is an inherent a limitation in understanding differences across racial/ethnic populations. Skeletal muscle changes should be investigated across more diverse patient populations in future investigations.

In addition to total skeletal muscle changes seen at T4, individual muscle regions such as PMM and EST were seen to have significant muscle composition reductions. Moreover, PMM was the only observed skeletal muscle group to experience area loss, thus indicating variability in rate of skeletal muscle loss by muscle group. One potential explanation for muscle specificity related losses may be skeletal muscle fiber typing. Under natural aging process, fast twitch muscle fibers are the first to be lost due to a shift from fast to slow twitch muscle fibers.^17^ Given that cancer treatment can mimic and accelerate physiological aging,^18^, ^19^, ^20^ it would be similarly anticipated that skeletal muscle groups with higher type II composition would likewise be the earliest to diminish. Previous studies have demonstrated that skeletal muscle mass loss due to chemotherapy arises primarily from atrophy of type IIb fast twitch fibers,^21^ whereas muscle groups which are known to be predominantly slow twitch or type I muscle fibers may be less vulnerable to atrophic conditions.^22^ Moreover, type I muscle fibers have been reported to be greatly affected by inactivity whereas type II fibers are more affected by diseases such as cancer.^17^, ^22^ During early cancer treatment, TRM did not see any significant area or composition loss in the AYA cohort. Similarly, EST did not have significant area loss. However, a reduction in EST composition was found to occur by Scan 2. Due to the highly variable nature of skeletal muscle composition,^23^ further studies are needed to better understand the relationship of anthracycline chemotherapy and muscle loss specificity.

AYAs experienced significant skeletal muscle area loss by Scan 2 irrespective of cancer type. However, only individuals diagnosed with sarcoma saw significant skeletal muscle index loss at 1 year. Sustained skeletal muscle loss in sarcoma patients may indicate a need for early prevention intervention based on type of cancer diagnosis. Sarcoma patients most often have extremity tumors which require surgery and/or radiation^24^ which may affect skeletal muscle function and mobility. Within the present cohort, approximately 45.5% of patients with sarcoma had lower extremity primary tumors. The combined effect of both the disease itself and lower limb treatments may explain the delayed skeletal muscle recovery among the cohort at 1 year.^25^ Although skeletal muscle loss is commonly reported across many cancer diagnoses,^26^ identifying patients with diagnoses which may be more greatly impacted or have increased recovery time may be advantageous for prevention of skeletal muscle deterioration and loss. Poor skeletal muscle status is known to directly impact clinical outcomes and have long term detrimental effects on cardiovascular health, metabolic health, and physical functioning.^27^, ^28^

Male AYAs experienced significant skeletal muscle area loss by Scan 2 whereas female AYAs did not. However, only female AYAs experienced significant changes in skeletal muscle quality as in contrast to males who did not experience a change in muscle quality after Scan 2. Previously conducted meta‐analysis have revealed that a skeletal muscle mass loss was 1.6 times higher in males than females.^29^ Differential skeletal muscle response during treatment should be further investigated to delineate hormone and endocrine response and the mediating role they may play in skeletal muscle status during cancer treatment.^30^

No clear or consistent pattern was observed when identifying skeletal muscle change by treatment type. We hypothesized that individuals who received radiation, high dose DOX, or combination of three treatment therapies (DOX + Radiation + Surgery) would experience greatest skeletal muscle loss. However, skeletal muscle reductions were experienced by almost all groups irrespective of treatment type grouping by Scan 2. Although, not all reached statistical significance. T4 skeletal muscle area loss was seen significantly in individuals who did not have radiation and individuals who received ≤300 mg/m^2^ DOX total dosage. Thus, suggesting that patients treated with anthracycline chemotherapy alone at any dose are at risk for skeletal muscle area loss as well. However, individuals who received radiation in addition to anthracycline chemotherapy experienced significant skeletal muscle composition reductions. This finding may suggest that radiation may have a greater deleterious impact on skeletal muscle quality rather than area. These findings should be further validated and may be additionally explained by the inability to control for additional confounding factors within the heterogenous treatment subgroups. Furthermore, factors such as hospitalizations, tumor location, nausea, vomiting, physical activity level, and nutrition intake were not factored into treatment grouping. Therefore, treatment classification alone in this case was not an adequate identifier of individuals who may experience significant skeletal muscle loss and future studies should be wary.

Alternative explanations behind unexpected SMI and SMD findings may be due to variability within CT characteristics. Although the CT scans utilized for the analysis were relatively consistent for voltage (120 kVp) and slice thickness (2.5–3.75 mm), additional parameters such as contrast and phase were more variable. Given that the nature of this study is retrospective, analysis was limited to the utilization of pre‐existing CT scans which were a part of routine disease status and treatment surveillance. Therefore, a limitation to the present study is that CT scans were unable to be requested, altered, or transformed from the pre‐existing conditions of the original scans. Due to this limitation, it is important to note the potential impact of inconsistent CT parameters to alter outcomes such as skeletal muscle index and density.^31^

Given the heterogeneity of the patient population, the impact of anthracycline therapy alone often cannot be assessed as most AYAs receive multiagent chemotherapy. However, we can infer from our mouse investigations that DOX therapy results in vascular compromise and decreased blood flow, resulting in presumed hypoxia, increased acidosis, and a decrease in muscle mass. Together, these have a deleterious impact on skeletal muscle loss. We demonstrated that 2 weeks of DOX therapy resulted in compromised skeletal muscle blood flow. Feed arteries and primary arterioles control total blood flow entering skeletal muscle.^32^ From feed arteries, microvascular units supply blood to the appropriate myofibers of the muscle.^33^ Blood flow to active skeletal muscle has been shown to be reduced with aging and subsequently results in a decreased in exercise capacity.^32^, ^34^ Physiological aging may be comparable to reductions in skeletal muscle blood after DOX administration and therefore provide key insight to DOX‐induced peripheral vascular changes and the impact on skeletal muscle. Similar to Gilliam et al.,^35^ we found that DOX therapy affected skeletal muscle morphology, collagen deposition, and apoptosis in both the gastrocnemius and soleus muscles. Soleus and gastrocnemius muscles additionally saw significant muscle fiber size reductions after treatment with DOX. Furthermore, skeletal muscle tissues showed the elevation of collagen deposition, indicating the early development of fibrosis. Fibrosis not only impairs muscle function; it is a major cause of muscle weakness and increases likelihood of re‐injury.^36^ Fibrosis is commonly seen in aging, muscular dystrophies, and severe injury.^36^ These functional and anatomic findings indicate that DOX therapy has the potential to induce skeletal muscle atrophy but also alter skeletal muscle composition and decrease muscular function via various pathways.

### 4.1 Conclusions

Early skeletal muscle loss occurred in approximately half of AYAs who were diagnosed with lymphoma or sarcoma during the first year of treatment. Skeletal muscle loss was significantly variable between skeletal muscle groups as well as patient characteristics such as sex and cancer type. Doxorubicin induced both reductions in skeletal muscle blood flow, skeletal muscle atrophy, and a change in skeletal muscle composition change in a mouse model. Understanding and early identification of skeletal muscle wasting in AYA patient with cancer is vital to the universal effort of preventing future co‐morbidities and early mortality associated with muscle loss during survivorship. Future studies are needed to aid in the continued effort of early identification of common characteristics, factors, and/ or biomarkers in AYA Hodgkin lymphoma and sarcoma patients who are at greatest risk of skeletal muscle loss while simultaneously providing direction for the development of targeted interventions in the identified at risk population aimed at preventing late effects.

## AUTHOR CONTRIBUTIONS

**Savannah V. Wooten:** Conceptualization (equal); data curation (equal); formal analysis (equal); investigation (equal); methodology (equal); project administration (equal); validation (equal); visualization (equal); writing – original draft (equal); writing – review and editing (equal). **Fei Wang:** Data curation (equal); formal analysis (equal); investigation (equal); methodology (equal); project administration (equal); visualization (equal); writing – original draft (equal); writing – review and editing (equal). **Michael E. Roth:** Conceptualization (equal); investigation (equal); methodology (equal); supervision (equal); visualization (equal); writing – original draft (equal); writing – review and editing (equal). **Guanshu Liu:** Formal analysis (equal); validation (equal). **J. Andrew Livingston:** Investigation (equal); writing – review and editing (equal). **Behrang Amini:** Investigation (equal); methodology (equal); project administration (equal); supervision (equal); validation (equal); visualization (equal); writing – original draft (equal); writing – review and editing (equal). **Susan C. Gilchrist:** Conceptualization (equal); investigation (equal); methodology (equal); supervision (equal); writing – original draft (equal); writing – review and editing (equal). **Michelle Hildebrandt:** Conceptualization (equal); data curation (equal); formal analysis (equal); funding acquisition (equal); investigation (equal); methodology (equal); project administration (equal); resources (equal); supervision (equal); visualization (equal); writing – original draft (equal); writing – review and editing (equal). **Eugenie S. Kleinerman:** Conceptualization (equal); funding acquisition (equal); investigation (equal); methodology (equal); project administration (equal); resources (equal); supervision (equal); visualization (equal); writing – original draft (equal); writing – review and editing (equal).

## FUNDING INFORMATION

This work was supported by the Harry S. Moss Heart Trust Fund and the MD Anderson Cancer Survivorship Seed Fund. Dr. Wooten is partially supported by The Alan Ryden and The Sanders Pediatric Sarcoma Endowment Funds.

## CONFLICT OF INTEREST STATEMENT

The authors report there are no competing interest to declare.

## ETHICS AND INTEGRITY STATEMENTS

Ethical committee (IRB) approval was obtained for the conductance of this study. Procedures followed were in accordance with the ethical standards of the Helsinki Declaration of 1975, as revised in 1983. The authors have no conflict of interest to declare. The data that support the findings of this study are available from the corresponding author, SW, upon reasonable request. The authors have no conflicts of interest to disclose at this time. This work was supported by the Harry S. Moss Heart Trust Fund and the MD Anderson Cancer Survivorship Seed Fund. Dr. Wooten is partially supported by The Alan Ryden and The Sanders Pediatric Sarcoma Endowment Funds.

## Supporting information


Table S1.

## Data Availability

The data that support the findings of this study are available from the corresponding author, SW, upon reasonable request.
