# Supplementary material for: Early skeletal muscle loss in adolescent and young adult cancer patients treated with anthracycline chemotherapy
Source: Cancer Med. 2023 Oct 30;12(22):20798–809. doi: 10.1002/cam4.6646 (PMC10709738; doi:10.1002/cam4.6646)
Supplement: Supplementary file 1 — Table S1. [file CAM4-12-20798-s001.docx]

**Supplemental Figure**

**Supplemental Table 1.** Participant Cancer and Treatment Characteristics

|  | **Lymphoma,**  **n = 120** | **Sarcoma,**  **n= 33** | **p** |
| --- | --- | --- | --- |
| Males / Females, n | M= 51/ F= 69 | M= 13/ F= 17 | 0.93 |
| Age a Diagnosis, y | 24.0 (17.0, 39.0) | 30.0 (15.0, 38.0) | 0.07 |
| BMI at Diagnosis, kg/m^2^ | 26.0 (16.7, 46.5) | 26.1 (17.9, 38.2) | 0.72 |
| **Treatment** |  |  |  |
| Radiation | 73 | 14 |  |
| Radiation Dose, Gy | 30.6 (19.8, 51.6) | 50.0 (37.5, 66.0) | <0.01* |
| Chest Radiation Exposure | 64 | 1 |  |
| Heart Radiation Exposure | 62 | 1 |  |
| Stem Cell Transplant | 16 | N/A |  |
| Surgery | 0 | 29 |  |
| Amputation | 0 | 4 |  |
| Chemotherapy | 120 | 33 |  |
| **Primary Anthracycline, n** |  |  |  |
| ABVD | 90 | 0 |  |
| ASHAP | 0 | 0 |  |
| CHOP | 3 | 0 |  |
| Dox/Cisplat | 0 | 6 |  |
| Dox/Ifosf | 0 | 12 |  |
| Vinc/Dox/Ifosf | 0 | 6 |  |
| VAdriaC | 0 | 1 |  |
| Other | 27 | 5 |  |
| **Secondary Anthracycline, n** | 16 | 13 |  |
| **Total Anthracycline Dosage, mg/m^2^** | 300 (75.0, 400) | 383 (150, 900) | <0.01* |

ABVD (doxorubicin, bleomycin, vinblastine, and dacarbazine), CHOP (cyclophosphamide, doxorubicin, vincristine, and prednisone), Dox (Doxorubicin), Cisplat (Cisplatin), Ifosf (Ifosfamide), Vinc (Vincristine), VAdriaC (Vincristine, Doxorubicin and Cyclophosphamide). All data is presented as Median (min, max) or n. *significance p<0.05
